# An RNAi supplemented diet as a reverse genetics tool to control bluegreen aphid, a major pest of legumes

**DOI:** 10.1038/s41598-020-58442-4

**Published:** 2020-01-31

**Authors:** Silke Jacques, Jenny Reidy-Crofts, Jana Sperschneider, Lars G. Kamphuis, Ling-Ling Gao, Owain R. Edwards, Karam B. Singh

**Affiliations:** 1 Centre for Environment and Life Sciences, CSIRO Agriculture and Food, Floreat, WA 6014 Australia; 2 Curtin University, Centre for Crop and Disease Management, Bentley, WA 6102 Australia; 3 Biological Data Science Institute, The Australian National University, Canberra, ACT 2600 Australia; 4 Centre for Environment and Life Sciences, CSIRO Land and Water, Floreat, WA 6014 Australia

**Keywords:** Assay systems, RNAi

## Abstract

Aphids are important agricultural pests causing major yield losses worldwide. Since aphids can rapidly develop resistance to chemical insecticides there is an urgent need to find alternative aphid pest management strategies. Despite the economic importance of bluegreen aphid (*Acyrthosiphon kondoi*), very few genetic resources are available to expand our current understanding and help find viable control solutions. An artificial diet is a desirable non-invasive tool to enable the functional characterisation of genes in bluegreen aphid and discover candidate target genes for future use in RNA interference (RNAi) mediated crop protection against aphids. To date no artificial diet has been developed for bluegreen aphid, so we set out to develop a suitable diet by testing and optimising existing diets. Here, we describe an artificial diet for rearing bluegreen aphid and also provide a proof of concept for the supplementation of the diet with RNAi molecules targeting the salivary gland transcript *C002* and gap gene hunchback, resulting in bluegreen aphid mortality which has not yet been documented in this species. Managing this pest, for example via RNAi delivery through artificial feeding will be a major improvement to test bluegreen aphid candidate target genes for future pest control and gain significant insights into bluegreen aphid gene function.

## Introduction

Aphids are the most economically important sap-sucking insect pests worldwide. With over 4,000 species that cause yield and financial losses both from direct damage by feeding, thereby draining essential nutrients from the plant, and as major vectors for disease, transmitting over 50% of all plant viruses^1^. Their asexual fast reproduction rate and clonal nature (parthenogenesis) results in fast developing resistance against different classes of insecticides, thereby increasing the cost and making it difficult to control them^2^.

*Acyrthosiphon kondoj* Shinji or bluegreen aphid is a major legume pest worldwide with particularly widespread distributions in North-America, Asia and Oceania (Invasive Species Compendium, CABI, 2018 update). Significant progress has been made in the understanding of genetic resistance towards bluegreen aphid in the model legume *Medicago truncatula*^3^. Two resistance genes were mapped to coiled coil (CC) nucleotide binding sequence – leucine rich repeat (NBS-LRR) rich regions on chromosome 3 and confer resistance against bluegreen aphid. The single dominant gene *AKR* (*Acyrthosiphon kondoi* resistance) involves both antixenosis and phloem-specific antibiosis^4^ whilst the semi-dominant gene *AIN* (*Acyrthosiphon* induced necrosis) mediates necrotic lesion formation at the site of infestation^5^. Resistance against bluegreen aphid in *M. truncatula* involves the recruitment of the octadecanoid biosynthetic pathway and upregulation of downstream jasmonic acid responsive genes^6^. Possible early regulators of bluegreen aphid resistance were revealed by transcription factor profiling showing a subset of transcription factors responsive to bluegreen aphid infestation was induced only in resistant *M. truncatula* varieties and required the presence of AKR^7^.

Plant defense response against aphid predation is only one part of the complex interplay in plant-aphid interactions. The identification and characterization of aphid genes and effectors promoting virulence are essential to understand the molecular basis of aphid infestation success and the evolutionary arms race between aphids and their host plant^8,9^. One way to functionally characterize aphid genes and test factors affecting aphid performance is through an artificial diet^10^. With a chemically defined diet it is possible to rear aphids without the need for host plants and to test the roles of factors such as pH, amino acid composition and sucrose content^11–13^. As such, they became a popular tool and artificial diets were developed for some aphid species^14,15^. Moreover, aphid artificial feeding is a reliable non-invasive delivery method to test the effects of supplemented molecules and genes. Double-stranded RNA mediated gene silencing, known as RNA interference (RNAi) is not only an important reverse genetics tool to decipher gene function^16^, it is also considered a potential insect pest management approach^17,18^. For example, providing dsRNA through artificial diets can uncover the importance and the usability of specific dsRNA molecules and pinpoint target genes of insect pests^19–21^.

With no bluegreen aphid genome currently available, its closest relative and model organism *Acyrthosiphon pisum* or pea aphid serves as the aphid reference genome^22^. RNAi studies in pea aphid led to the discovery of essential genes that when silenced via dsRNA cause insect lethality. Injection of small interfering RNA (siRNA) targeting C002, the most abundant salivary gland transcript in pea aphid, resulted in silencing and greatly increased mortality rates of the injected insects^23^. Further experiments showed the C002 protein is secreted into the host plant during aphid feeding and is crucial for phloem sap ingestion^24^. Feeding based RNA interference of the *A. pisum* gap gene hunchback (*Aphb*) also significantly increased mortality rates^20^. These are only two examples of a range of RNAi target genes explored in pea aphid, others include a gut digestive enzyme *cathepsin-L*^25^, a structural sheath protein *SHP*^26^ and a chitin synthase gene (Ye *et al*., 2019). Targeting bacterial symbiosis-related genes of pea aphid by RNAi also showed potential as a novel strategy for controlling sap-feeding insect pests^27^. RNAi applications are not limited to pea aphid, with RNAi delivery established in other aphid species^28^, including cotton aphid^29–31^ and green peach aphid^32^. RNAi applications in other insect species have gained important traction and have contributed significantly to the advances in insect pest management^33^.

Despite the economic importance of bluegreen aphid there are very few genetic resources that are available to expand our current knowledge and to develop novel and viable bluegreen aphid control strategies. An artificial diet could enable the functional characterisation of genes in bluegreen aphid and uncover candidate gene targets for future use in RNAi-mediated crop protection against bluegreen aphids. Here, we formulated a chemically defined diet suitable for bluegreen aphid to feed and reproduce. By testing and adapting previously reported diets and minimizing space occupancy of the feeding cages, we delivered an optimized platform which can be implemented as a fast and easy tool to better understand the molecular basis of aphid feeding and pathogenicity. By supplementing the artificial diet with RNAi molecules, we highlight the potential to employ this diet as a quick and effective reverse genetic approach for bluegreen aphid. We are confident this approach is a major breakthrough to uncover candidate RNAi target genes and effectors for future pest control of bluegreen aphid.

## Results

### An artificial diet for bluegreen aphid

**Figure 1:**
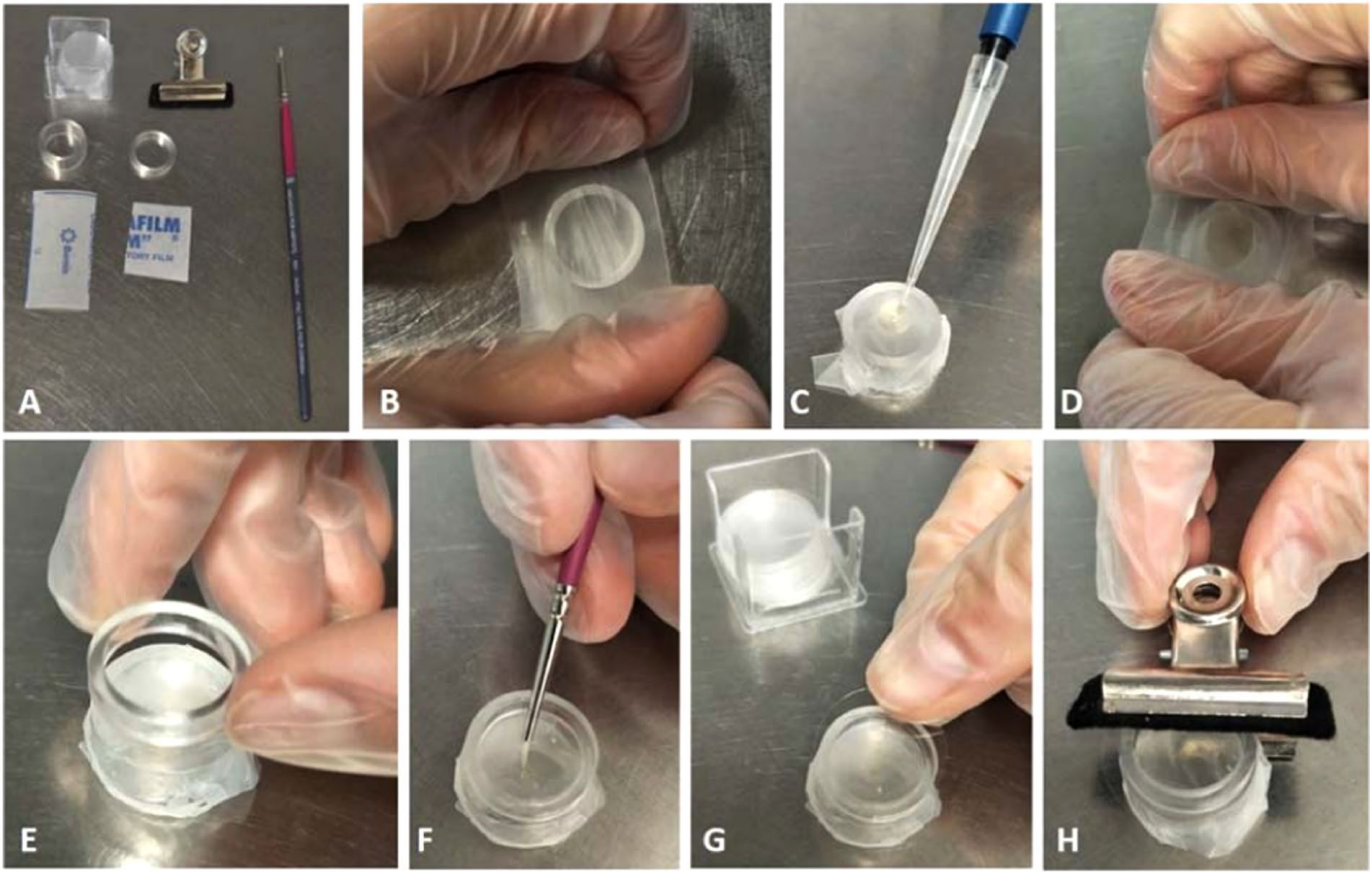
Assembly of a diet cage. Two sheets of parafilm, two plexiglass rings, a paintbrush, metal clip and coverglass are needed to prepare a diet cage. (**A**) The larger parafilm sheet is stretched over the 1 cm plexiglass ring (**B**) and artificial diet is loaded onto the membrane. (**C**) The second parafilm sheet is stretched out over the diet so it is completely covered, thereby trapping it and forming a feeding pouch. (**D**) Aphids are transferred with a fine paintbrush and are held in place with a second plexiglass ring. (**E,F**) Finally, the diet cage is sealed with a coverglass secured by a metal clip (**G,H**).

**Figure 2:**
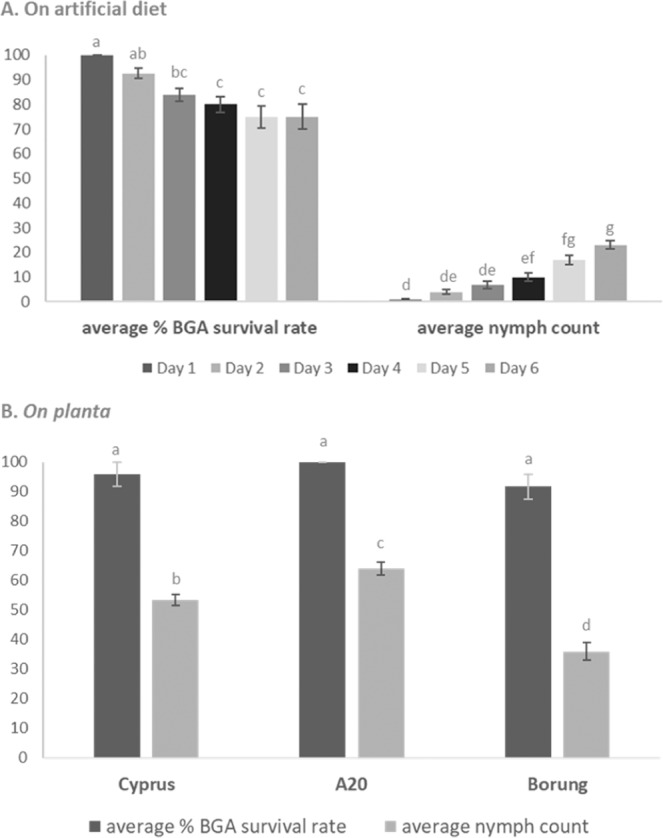
Bluegreen aphid can feed and reproduce on the artificial diet. (**A**) A total of ten cages with eight bluegreen aphid adults per cage were monitored for six days and BGA mortality and nymph production was recorded. The average survival and nymph production rates are shown with their respective standard error. From day five, the diet in three cages was contaminated and this number increased to six cages on day six. Aphid numbers on non-contaminated diets remained stable. From day three onwards, there were no significant differences in bluegreen aphid survival rates, nor was there a significant difference between day two and day three (Tukey-Kramer multiple comparison test, *P* < 0.05) demonstrating overall stable numbers of bluegreen aphid reared on the artificial diet. Aphid nymph numbers increased over time with a significant increase from day four onwards (ANOVA with Tukey-Kramer multiple comparison post-hoc test, *P* < 0.05, different letters indicate significant differences between groups). (**B**) Bluegreen aphid adults were allowed to feed on a trifoliate leaf of three susceptible *M. truncatula* cultivars, Cyprus, A20 and Borung. Bluegreen aphid mortality and nymph production was recorded after six days of feeding. No significant survival differences are observed between the three cultivars whilst the nymph production rates are dependent on the cultivar with the highest number of nymphs produced on A20 (ANOVA with Tukey-Kramer multiple comparison post-hoc test, *P* < 0.05).

Aim of this study was to develop an effective artificial diet for bluegreen aphid. As the model organism *Acyrthosiphon pisum* is the closest relative to bluegreen aphid with a reference genome available, we first tested the feasibility of the pea aphid diet to rear and study bluegreen aphid. The artificial diet was provided in a cage feeding platform optimized to minimize space occupancy compared to performing whole plant studies in the glasshouse. A step by step preparation of the feeding cage and diet loading is depicted in Fig. 1. Loading the artificial diet between parafilm sheets allowed for bluegreen aphid to puncture the stretched membrane with its stylet and to feed on the provided diet. Both the chemically defined diet described by Auclair & Cartier^34^ and the improved aromatic amino acid balance^35^ of the pea aphid artificial diet were not suitable to rear bluegreen aphid as the aphids did not settle on the diet (data not shown). Next, the synthetic diet that allowed a permanent culture of *Myzus persicae* to be generated^10^ was trialed but was also not suitable and showed a mortality rate greater than 50% within 48 hrs. By increasing the sucrose concentration from 15% to 17.5% bluegreen aphid survival rates climbed to 80% after two days (Supplementary Table 1). Addition of cholesterol (2.5 mg) and reduction of manganese sequestrene concentration by half (0.4 mg) recovered average survival rate of bluegreen aphid to 92% 48 hours after feeding on the diet (Fig. 2). The final composition of the diet is provided in Supplementary Table 2 with changes to the Dadd & Mittler diet^10^ highlighted with an asterisk.

To further characterize the use of the artificial diet, a total of eighty aphids divided over ten cages were followed for six days and mortality and nymph production was recorded daily (Fig. 2). Although the assembly of diet cages was prepared in the laminar flow, from day five onwards several diets showed microbial contamination promoting bacterial and fungal growth and thereby killing off the aphids. However, aphid numbers on non-contaminated diets remained stable and an average of 75% survived for six days. A Tukey-Kramer multiple comparison test showed survival rates at day three are not significantly different from day two, four, five and six (*P* < 0.05) highlighting the stability of this diet over time (Fig. 2A). Production of nymphs reaching a maximum on day six with an average of 23 nymphs per cage, showing aphids settle well on the diet. Aphid nymph numbers increased significantly from day four onwards (*P* < 0.05, Fig. 2A). To gain an understanding how well the aphids perform on an artificial diet compared to natural feeding *on planta*, we included three *M. truncatula* lines (A20, Cyprus and Borung), all susceptible to bluegreen aphid and scored the aphid survival rates and nymph count after six days of feeding (Fig. 2B). An additional reference genotype of *M. truncatula*, A20, is highly susceptible to bluegreen aphid and contains neither AKR nor AIN^36^. Cyprus and Borung are both susceptible lines also lacking AIN, and resistant near isogenic lines for each of these cultivars were generated to study resistance to bluegreen aphid and spotted alfalfa aphid^37^. No significant differences in aphid survival rates were observed between the three *M. truncatula* cultivars after six days of feeding on a trifoliate leaf (*P* < 0.05, Fig. 2B). The highly susceptible A20 line has 100% bluegreen aphid survival rate whilst on Cyprus and Borung 96% and 92% of aphids survive, respectively. Nymph production was significant between cultivars with an average of 64 nymphs on A20 plants as the highest nymph count, followed by Cyprus with 53 nymphs and Borung with an average of 36 nymphs produced after six days (Fig. 2B). Whilst the survival rate and nymph count is higher *on planta* compared to *in vitro*, our data shows that bluegreen aphid is able to feed and produce nymphs (Fig. 2A) on modified artificial diet (Supplementary Table 2) in an optimized cage feeding platform (Fig. 1).

### Finding conserved motifs of lethal candidate genes

**Figure 3:**
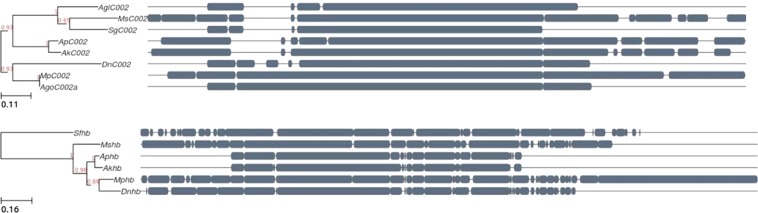
Phylogenetic tree and sequence alignment of Aphbd and ApC002 genes in different aphid species. Multiple sequence alignment of the homologous aphid sequences of the *Acyrthosiphon pisum C002* (*ApC002*) and hunchback gene (*Aphbd*) show these sequences are highly conserved. The phylogenetic tree confirms that *A. pisum* is bluegreen aphid’s closest relative. Branch support values are highlighted in red. Abbreviations: *Agl* = *Aphis glycines; Ms* = *Melanaphis sacchari; Sg* = *Schizaphis graminum; Ap* = *Acyrthosiphon pisum; Ak* = *Acyrthosiphon kondoi; Dn* = *Diuraphis noxia; Mp* = *Myzus persicae; Ago* = *Aphis gossypii; Sf* = *Sipha flava*.

To provide a proof of concept that this diet when combined with RNAi strategies can be used as an effective reverse genetics approach, we looked for target genes that were previously reported to be lethal to *A. pisum*, a close relative of the same genus to bluegreen aphid, when knocked down via RNAi. We focused on two pea aphid targets, the salivary gland transcript *C002* and the gap gene hunchback *Aphbd*, which resulted in aphid mortality when knocked down by injected targeted siRNA or feeding-based dsRNA, respectively^20,23^. Based on literature review and BLAST analysis, six sequences homologous to *C002* and four *Aphbd* homologous sequences in different aphid species were found. The sugarcane aphid (*Melanaphis sacchari*), green peach aphid (*Myzus persicae*) and Russian wheat aphid (*Diuraphis noxia*) have homologs to both the salivary gland and hunchback gene whilst the soybean aphid (*Aphis glycines*), wheat aphid (*Schizaphis graminum*) and cotton aphid (*Aphis gossypii*) only contributed a *C002* homolog and the yellow sugarcane aphid (*Sipha flava*) an *Aphbd* homolog. The bluegreen aphid homologous sequences were obtained from a preliminary *de novo* assembled *A. kondoi* draft genome available in-house and will henceforth be referred to as *AkC002* and *Akhbd* (Supplementary Figure 1). A total of eight *C002* and six hunchback gene sequences were aligned using ClustalW and were used to construct a phylogenetic tree based on nucleic acid sequences (Fig. 3). The tree confirms that pea aphid is the closest relative to bluegreen aphid and shows high conservation of the candidate genes. For the synthesis of double stranded RNA, T7 appended primers were designed to target a highly conserved region across the retrieved aphid sequences of the *AkC002* and *Akhbd* (Supplementary Fig. 2).

### RNAi mediated gene silencing via the bluegreen aphid diet

Double stranded RNA molecules were supplemented to the artificial diet to test the feasibility of RNAi feeding bioassays for bluegreen aphid. Previous reports of our selected target genes, *ApC002* and *Aphbd*, showed a significant increase in pea aphid mortality three days after RNAi mediated gene silencing^20,23^. As we required a fast system allowing for future high-throughput screening of target gene candidates in in bluegreen aphid, we followed mortality rates and nymph production for three days. Moreover, there was no significant difference in bluegreen aphid survival rate on the artificial diet between day two and three and from day three onwards (Tukey-Kramer multiple comparison test, *P* < 0.05, Fig. 2).

**Figure 4:**
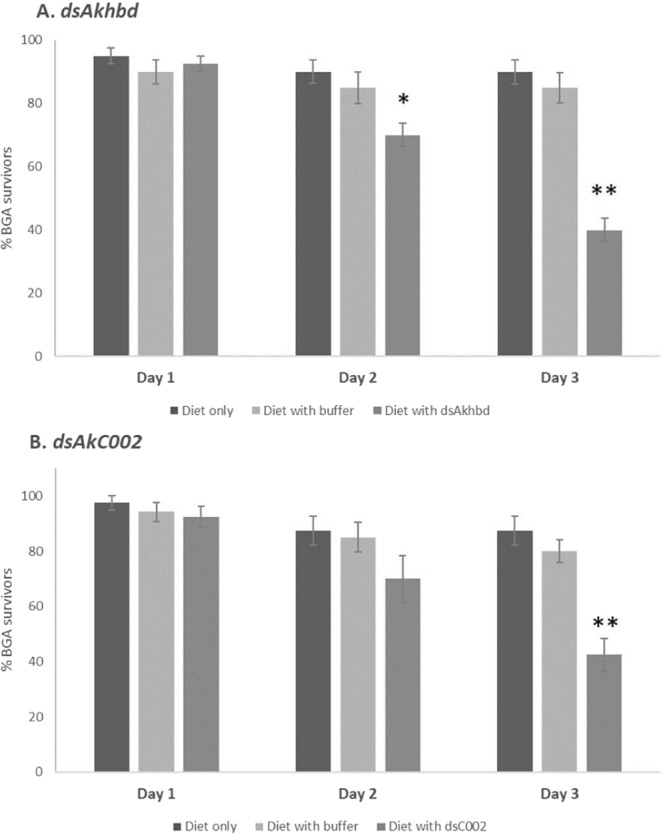
dsRNA supplemented diet is lethal to bluegreen aphid. Bluegreen aphid feeding bioassays were followed for three days and aphid mortality was recorded. Cages either contained diet only (black bars), diet mixed with elution buffer (light grey bars) or diet mixed with dsRNA (dark grey bars) targeting *Akhbd* (**A**) or *AkC002* (**B**). Feeding on dsRNA supplemented diet significantly decreases survival rate after three days (*P* < 0.01, Mann-Whitney U test) compared to both controls (shown as double asterisk). For *dsAkhbd*, bluegreen aphid mortality significantly increases already after two days compared to aphids feeding on the diet only control (*P* < 0.01, ANOVA) indicated by a single asterisk (**A**).

The amplified *Akhbd* target sequence was 503 bp in size whilst the *AkC002* was 626 bp, not including the T7 sequence. Subsequent synthesis of dsRNA with the MEGAscript® RNAi Kit yielded pure and good quality RNAi molecules. However, mixing dsRNA eluted in extraction buffer with the artificial diet might have a negative impact on the carefully balanced diet and as such influence bluegreen aphid fitness. Therefore, to exclude any negative effect coming from the elution buffer on bluegreen aphid survival rate and nymph production, we introduced a diet mixed with elution buffer only as an additional control. Hence, we now tested three conditions: artificial diet, artificial diet with elution buffer only, artificial diet with elution buffer containing dsRNA. In a first batch of experiments, 18 µL of dsRNA eluted in extraction buffer was mixed with the diet to reach a final concentration of 50 µg/µL. Feeding assays with control diet mixed with this amount of elution buffer decreased nymph production numbers and also reduced bluegreen aphid survival rate, but not as pronounced as the dsRNA supplemented diet did (data not shown). Consequently, we optimized the protocol by concentrating eluted dsRNA using vacuum centrifugation. This reduced the volume of 18 µl dsRNA to 5 µL for *dsAkhbd* and 10 µL for *dsAkC002* to reach final concentrations of 63 µg/µL and 51 µg/µL respectively. These volumes of elution buffer addition did not result in a significant difference in survival rate with bluegreen aphid feeding on diet only (*P* < 0.05, Fig. 4). On the other hand, the feeding bioassays with *dsC002* or *dsAkhbd* negatively impacted survival rates and lead to a significant increase in mortality after three days of feeding (*P* < 0.01, Fig. 4). Even after just two days, the diet mixed with dsRNA targeting *Akhbd* significantly increased mortality (*P* < 0.05) in bluegreen aphid compared to control aphids (Fig. 4A). On day three an average of only 40% survived the artificial diet mixed with dsRNA targeting *Akhbd* compared to 82.5% feeding on artificial diet with elution buffer and 95% feeding on diet only (Fig. 4B). Comparable results were obtained with *dsAkC002* supplemented diet with bluegreen aphid survival rate dropping to 42.5% after three days whilst control aphids feeding on diet only and diet with buffer were stable at 87.5% and 80% respectively (Fig. 4A). In addition, we performed survival analysis using the Kaplan-Meier curves and log ranking testing to show the survival rates are significantly different between treatments (Supplementary Fig. 2). The addition of *dsAkhbd* (A) and *dsC002* (B) to the diet resulted in highly significant Kaplan-Meier survival curves (*P* < 0.0001).

**Figure 5:**
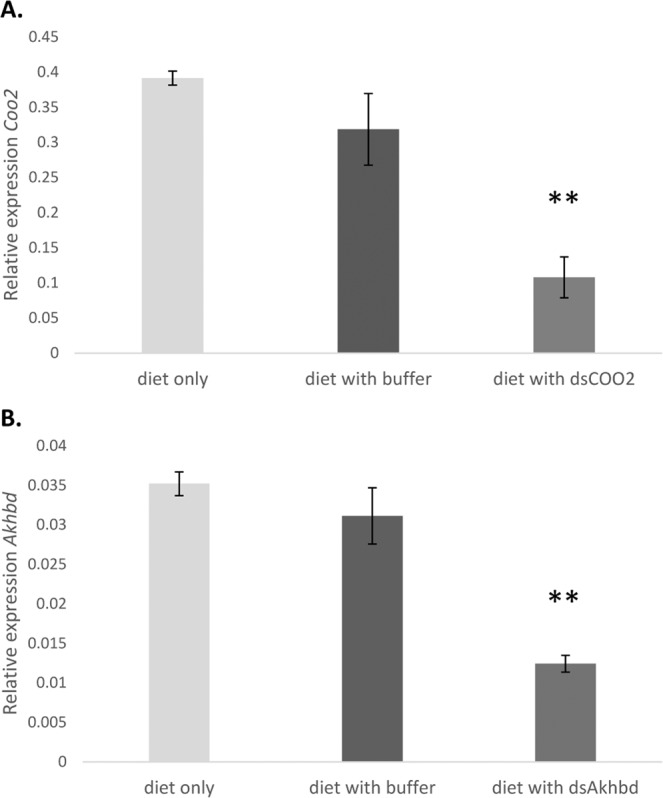
Down-regulation of target gene upon dsRNA uptake. Relative expression of the target genes *C002* (**A**) and *Akhbd* (**B**) in bluegreen aphids was recorded by qRT-PCR after two days of feeding on diet cages with diet only (light grey), diet mixed with elution buffer (black) or diet supplemented with dsRNA (dark grey). Whilst the buffer had no significant effect, dsRNA uptake by the aphids significantly lowered the relative expression of its respective target gene; *C002* (**A**) and *Akhbd* (**B**) (*P* < 0.01, ANOVA).

To test whether the dsRNA uptake results in a down-regulation of its target gene, a follow-up quantitative real-time PCR (qRT-PCR) was conducted with surviving aphids after two days of feeding. Bluegreen aphids feeding on diet with elution buffer has no effect on the relative expression of the target genes *C002* and *Akhbd* whilst the diet supplemented with dsRNA results in a significant down-regulation of its respective target gene (*P* < 0.01) (Fig. 5). Taken together, these results provide a proof of concept for successful supplementation of the bluegreen aphid artificial diet with RNAi molecules targeting two candidate genes that resulted in bluegreen aphid mortality.

## Discussion

Aim of this study was to develop an artificial diet for bluegreen aphid to employ as an effective reverse genetics tool to rapidly screen for candidate aphid genes that play an essential role in either aphid feeding or pathogenicity. Here, we provide an optimized diet and a small cage feeding platform, including the use of relatively small quantities of artificial diet (100 µL) where bluegreen aphids settle, feed and produce nymphs. We further showed the promising potential of this diet to uncover potential candidate genes for future use in pest control management. Through supplementation of double stranded RNA molecules in the diet, we delivered the first report of RNA interference mediated uptake in bluegreen aphid and showed that ingestion of *dsAkC002* and *dsAkhbd* resulted in bluegreen aphid mortality and down-regulation of their respective target genes.

We succeeded in developing an artificial diet to enhance our understanding of the feeding and pathogenicity of bluegreen aphid (Supplementary Table 2). In 1946, the first multicellular organism (*Drosophila melanogaster*) was reared on a chemically defined diet^38^ but it wasn’t until 16 years later that the first diet for the sap-sucking green peach aphid (*Myzus persicae*) was defined^39^. The formulation of an artificial diet to rear pea aphid quickly followed^34^. We initially tested this original pea aphid synthetic diet as well as the diet with an optimized amino acid balance^35^ but neither were suitable for bluegreen aphid as the aphids did not settle. So even though pea aphid is the closest relative to bluegreen aphid and their host range partially overlaps as both species can infest lucerne, clover and faba bean, they cannot feed on the same artificial diet. Next, we trialed the green peach aphid diet composition^39^ which was also not optimized for rearing bluegreen aphid and survival numbers were reduced by half within 48 hours. Thus, we evaluated the green peach aphid artificial diet and started modulating factors known as phagostimulants which induce insects to ingest and effectuate sustained feeding^40^. Sugars are a main feeding stimulant and sucrose in particular was shown to be phagostimulatory to aphids^41^. Indeed, an increase in sucrose concentration from 15% to 17.5% significantly lifted bluegreen aphid survival rates from less than 50% to 80% within 48 hours. Survival rates were increased further by adding cholesterol, a known phagostimulant for various insects, and previously reported to significantly extend aphid lifespan and increase fecundity^42^. The report focusing on optimal levels of trace metals in combination with cholesterol^43^ led us to reduce the manganese concentration to half the zinc concentration which led to a further increase in survivorship and resulted in an average of 92% bluegreen aphid after two days. Compared to 75% of bluegreen aphids surviving after six days of feeding on our newly developed artificial diet (Fig. 2A), only 50% of green peach aphids survived after six days on an 18% sucrose solution or after thirteen days on a complete synthetic diet^39^. The average nymphs produced per adult is also higher on our diet at 23 nymphs per adult compared to 19 on the green peach artificial diet. The bluegreen aphid survivorship after six days (75%) falls in the range of first-instar pea aphid nymphs surviving on four modified artificial diets for seven days (50% to 90% survivorship)^34^. Whilst aphids feeding *on planta* are more stable and produce more nymphs, this is to be expected from an artificial system versus a natural feeding environment (Fig. 2). The susceptible *M. truncatula* cultivar Borung was closest to the artificial diet with a bluegreen aphid nymph count of 36 compared to 23 on the artificial diet. Despite limited space in the cage feeding platform and contamination of the diet from day five onwards, the bluegreen aphids settled well on the diet and had a stable survivorship of 75% after six days of feeding.

The successfully modified artificial diet for rearing bluegreen aphid is advantageous to study aphid behaviour since the exact chemical composition of the diet is known and remains consistent. In host plants, on the other hand, aphids are reared on the plant’s phloem composition, which is subject to environmental conditions (light, temperature, etc.) and can therefore vary. Aphids reared in small feeding cages also require less space compared to whole plants. Moreover, an artificial diet allows for the easy introduction or removal of molecules, making it an ideal system to study aphid gene function and discover candidate effectors. Our newly developed artificial diet suitable for bluegreen aphid can now be used as part of a reverse genetics platform to find and characterize aphid target genes.

RNA interference (RNAi) is a post-transcriptional gene silencing mechanism that can be used to trigger sequence specific degradation of candidate genes^16^. Targeted gene knockdown by dsRNA-mediated RNAi has been extensively used to study gene function in insects, including aphids, and has been reviewed in detail^17^. Delivery methods for dsRNA in aphids are through direct micro-injection or oral administration, either diet-based or via feeding on transgenic plants. Potential RNAi target genes have been explored in multiple aphid species including pea aphid^20,24,25,32^, cotton aphid^29–31^ and green peach aphid^32^. Although bluegreen aphid causes major damage to grain legumes such as lupins and to various leguminous pastures such as lucerne and clover, no RNAi studies were reported to date for bluegreen aphid. The relatively small size of this aphid compared to pea aphid is a limiting factor for RNAi delivery through direct injection as this would induce mechanical damage. Therefore, feeding based administration of dsRNA was desirable as this provides a high-throughput strategy to test candidate target genes and can be easily translated for use in the field^44^.

To provide a proof of concept, we focused on two clear RNAi target genes reported to be lethal in pea aphid, the model organism and closest relative to bluegreen aphid. Injection of short interference RNA (siRNA) targeting the salivary protein ApC002 causes a significant decrease in *ApC002* transcript levels over a three day period which was reflected in only a 50% survival rate of pea aphid after three days^23^. Our results showed a 42% bluegreen aphid survival rate after three days of feeding on artificial diet supplemented with dsRNA targeting the homologous *ApC002* gene in bluegreen aphid compared to 87.5% survival rate when feeding on diet only (Fig. 4). This corresponded to a significant down-regulation of the *C002* transcript when feeding on diet supplementation with *dsC002* (Fig. 5A). This suggests an even higher efficiency of feeding based RNAi mediated gene silencing compared to delivery via direct injection in pea aphid^23^. When green peach aphid (*Myzus persicae*) fed on tobacco leaf disks transiently producing dsRNA targeting the *MpC002* gene, its partial silencing significantly reduced aphid reproduction rate, but not survival rate^28^. This was in contrast to the findings in wheat aphid (*Schizaphis graminum*) where a complete knock-down of the *SgC002* gene could cause high mortality of aphids^45^. This illustrates how silencing of homologous genes can vary significantly between different aphid species and is dependent on a multitude of factors, including the structural accessibility of the target site^46^. Other factors such as the effective dosage and length of the RNAi construct are important differences that need to be taken into consideration to induce optimal silencing in the target organism^47^. We opted to supplement the artificial diet with long dsRNA since this has the benefit of generating multiple siRNA targeting the candidate gene compared to providing a single siRNA molecule. For example, comparison of RNAi molecules in the flour beetle *Tribolium castaneum* showed that a single siRNA was less efficient than a long dsRNA for the same target gene with the siRNA hardly triggering any phenotypic changes for intact insects^48^.

The second target gene was the gap gene hunchback whose silencing through feeding supplemented dsRNA significantly raised mortality in pea aphid after three days compared to controls^20^. The average survival rates of bluegreen aphid were as low as 40% compared to 85% in control diet after three days feeding on artificial diet with dsRNA designed against the conserved downstream segment of the hunchback homolog in bluegreen aphid (*Akhbd*) (Fig. 4). This is reflected in the down-regulation of the *Akhbd* transcript compared to the controls (Fig. 5B). Mortality rates observed in artificial feeding bioassays can differ between target organisms and are dependent on a range of factors such as varying RNAi efficiencies in different target organisms, concentration of the RNAi molecules supplemented in the diet and daily renewal of the diet.

The successful application of feeding based RNAi in bluegreen aphid opens the possibility to screen for essential target genes in bluegreen aphid whose knockdown induces mortality or reduced fitness and performance. The diet can also be used for the identification of aphid effectors promoting pathogenicity which can help to develop novel strategies for pest resistances in plants. Our set-up allows for high-throughput screening as it is a fast turnover experiment, requires minimal space occupancy and is easy to implement. Improvements could be made to the elution buffer to isolate dsRNA as it also slightly reduces survival rates compared to bluegreen aphid feeding on diet only. Other dsRNA delivery methods in aphids should also be explored such as soaking or spraying^49^. A virus-based RNAi vector expressed in citrus tree induced gene silencing of a phloem-sap sucking insect *Diaphorina citri*^50^ and could be a way to hijack virus-carrying aphids.

This work forms the basis to include bluegreen aphid to the growing list of agricultural insect pests that could be managed through RNAi based control solutions^17,44,47^ with the generation of aphid-resistant wheat plants as the latest example of plant-mediated RNA interference to combat grain aphid^51^. This would reduce the need for chemical insecticide use and the future risk of losing insecticide efficacy due to the development of insecticide resistance as there are many potential aphid genes that could be targeted, either individually or in groups of two or more. The sequence specificity of RNAi mediated gene silencing allows one to target individual species thereby mitigating the risk of affecting beneficial organisms. Our study shows that potential target genes for bluegreen aphid pest control can now be tested in a fast and efficient way via the RNAi mediated artificial diet feeding platform for bluegreen aphid.

## Material and Methods

### Aphid rearing and host plants

*Acyrthosiphon kondoj* Shinji aphids were obtained from an asexual, parthenogenetic colony initiated from a single bluegreen aphid clone collected in Western Australia. Aphid numbers were maintained by rearing them on caged 4-week-old subclover (*Trifolium subterraneum*) cv. ‘Dalkeith’ plants in natural light in the greenhouse with temperatures ranging from 15 °C - 30 °C. Aphids were transferred to feeding cages with a fine paintbrush.

### Artificial diet preparation

The artificial diet by Dadd & Mittler^10^ was optimized to use as the bluegreen aphid artificial diet. The full list of components and final concentrations can be found in Supplementary Table 2. The diet was mixed in the following order: add the twenty amino acids first, followed by sucrose, then add the ten vitamins and six metal salts and add cholesterol last. A ten times stock solution is made for the vitamins D-biotin, folic acid and riboflavin, as for the cupric-sodium EDTA salt, zinc chloride and manganese chloride tetrahydrate since these are added in minute amounts. Final diet will have an initial pH of 4.35 and was adjusted to pH 7 by adding potassium hydroxide. The diet was filtered through a sterile 0.2 µm syringe filter (Thermo Fisher Scientific, Waltham, USA) and 8 ml aliquots were stored aseptically in sterile volumetric graduated thick 10 mL glass vials (DWK Life Sciences, Millville, USA) capped with rubber stoppers and sealed with aluminium cramping lid sealers. Diet vials can be stored at −20 °C or used immediately. Once defrosted, all the diet should be used as re-freezing will result in premature oxidation of the diet and promote bacterial growth.

### Feeding cage assembly

A Parafilm® sheet (2.5 cm by 5 cm) was stretched across a Plexiglass ring of 1 cm high (20 mm diameter) and 100 µL of artificial diet was pipetted on top of the Parafilm® layer. A second sheet of Parafilm® (2.5 cm by 2.5 cm) was stretched out over the droplet thereby trapping the diet and forming a feeding sachet penetrable by the aphids’ stylet. Aphids were confined to the sachets by placing another plexiglass ring of 3 mm high on top and sealing the top with a 21 mm diameter circular coverglass (Knittel, Bielefeld, Germany) secured with a padded metal clip (Esselte Letter Clips 31 mm). All cages were prepared in the laminar flow and aphids were transferred to the cage using a fine paintbrush.

### dsRNA synthesis of AkC002 and Akhbd

A 250 mg mix of adult and nymphs bluegreen aphid were ground to a fine powder under a stream of liquid nitrogen and DNA was extracted using the CTAB method as described previously in^52^. T7 appended primers were used to amplify a conserved region in *AkC002* and *Akhbd* with C002 forward primer 5′- TAATACGACTCACTATAGGGGGGAAGTTACAAATTATACG-3′ and reverse primer 5′-TAATACGACTCACTATAGGGCTCCCATAGCCATCTTG-3 and *Akhbd* forward primer sequence 5′-TAATACGACTCACTATAGGGAGTGGCGGTGAATTGACG-3′ and reverse primer 5′-TAATACGACTCACTATAGGGAACGGGTCCCTGAAGCT-3′. For each of the genes, a single PCR run was performed at 98 °C for 3 min, followed by 35 cycles of 98 °C for 15 s, 63 °C for 25 s, and 72 °C for 50 s, finishing with an extension step at 72 °C for 10 min. PCR products were run on a 1% agarose gel in TAE buffer for 40 min at 90 V to verify purity and size. Isolation of the PCR products was performed via excision of the single band of the expected size for each gene target and purified with the Qiaquick gel extraction kit (Qiagen, Hilden, Germany). Subsequent transcription reaction, nuclease digestion of DNA and ssRNA and purification of the dsRNA was performed using the Megascript RNAi Kit (Ambion, Texas, USA) in accordance with the provided protocol guidelines. The purified dsRNA was quantified with a spectrophotometer at 260 mm and checked for size, purity and integrity on an agarose gel.

### Development of phylogenetic tree to identify homologous target genes

A nucleotide BLAST search of the pea aphid full length salivary gland transcript *C002* and the pea aphid hunchback gene - downstream *Aphbd* was performed against the nucleotide collection and optimized to find highly similar sequences (megablast). An additional nucleotide BLAST search against the in-house available full length *AkC002* and *Akhbd* sequences was performed using the same parameters. All homologous aphid *C002* and hunchback DNA sequences were extracted from NCBI in fasta format. A multiple sequence alignment of eight *C002* sequences and six hunchback sequences was performed using Clustal Omega^53^. Phylogenetic trees were predicted using the phyton framework ETE3 build command^54^ with the RaxML (randomized axelerated maximum likelihood) workflow^55^ to estimate the maximum likelihood phylogeny.

### Feeding bioassays for bluegreen aphid

To test the suitability of a specific diet, eight apterous bluegreen aphid adults were placed onto the feeding sachet and a total of ten biological repeats were performed over six days. Survivor rates were noted as well as nymph count. A mixture of early and late instar nymphs was also tested on the artificial diet using five replicates of five nymphs. Ten biological repeats were used for the Tukey-Kramer multiple comparisons test in NCSS12 to calculate all pairs (days) simultaneous confidence intervals of mean difference and *P*-value (α = 0.05). For the *on planta* feeding comparison, eight apterous bluegreen aphid adults were placed on a single fully expanded trifoliate leaf from the primary stem of individual four-week-old plants and contained in a socket cage as described previously^37^. Three susceptible *M. truncatula* cultivars were used, A20, Borung and Cyprus and were acquired from the Genetic Resource Centre, the South Australian Research and Development Institute (SARDI). Seeds were scarified, bleached and germinated and plants were grown as described by Klingler and colleagues^4^. Six biological replicates were set up for each aphid‐infested line and ANOVA with post-hoc Tukey-Kramer multiple comparisons test was performed to identify significance (*P* < 0.05).

RNAi feeding assays consisted of artificial diet supplemented with 5 µL dsRNA targeting *Akhbd* or 10 µL dsRNA to target *AkC002* to a final concentration of 63.5 µg/µL and 50.6 µg/µL respectively. A biological repeat consisted of eight cages per treatment (artificial diet only; artificial diet + *dsAkC002*; artificial diet + *dsAkhbd*; artificial diet + dsRNA elution buffer), each containing five adult apterae and mortality rates were checked daily for three days. The initial RNAi feeding bioassay targeting *AkC002* and *Akhbd* were tested in separate trials and controls containing only diet and diet with dsRNA elution buffer were included in each. A total of three biological repeats (each repeat consisting of eight cages per treatment, 5 adults per cage) was used for Kaplan-Meier survival curves and log-rank testing (*P* < 0.0001) using GraphPad Prism Statistical Software program (Supplementary Fig. 2) as well as ANOVA and post-hoc Tukey-Kramer multiple comparisons test (*P* < 0.05 and *P* < 0.01) (Fig. 4).

A repeat RNAi feeding bioassay for follow-up qRT-PCR consisted of artificial diet supplemented with 10 µL freshly made dsRNA targeting *Akhbd* or *AkC002* to their respective final concentrations of 65.6 and 49.9 µg/µL. A total of fifteen cages per treatment were set-up and alive aphids were collected for subsequent RNA extraction after two days of feeding on either diet only, diet with 10 µL elution buffer, diet with 10 µL *dsC002* or diet with 10 µL *dsAkhbd*.

All feeding cages were incubated at a constant temperature of 20 °C in a growth cabinet (Percival Scientific, Iowa, US) using a 14:10 h light: dark cycle.

### qRT-qPCR

All aphids still alive after two days of feeding were transferred with a fine paintbrush to 1.5 mL Eppendorf tubes and aphids of five feeding cages per treatment (diet only; diet with elution buffer; diet with *dsAkC002*; diet with *dsAkhbd*) were pooled to form one biological repeat. The aphids were frozen in liquid nitrogen and ground to a fine powder for subsequent RNA isolation using the RNeasy Plus Mini Kit (Qiagen, Hilden, Germany) according to manufacturer’s instructions. RNA was eluted in 30 µL of RNase free water and concentrations were measured using Nanodrop (Thermo Fisher Scientific, MA, USA). A total of 1 µg of total RNA was used for first-strand cDNA synthesized according to the Superscript^TM^ III reverse transcriptase manual (Invitrogen, CA, USA). qRT-PCR was performed using a 384 iCycler (BioRad, CA, USA) using SES mastermix according to manufacturer’s instructions (Biorad, CA, USA) and thermocycling conditions as described previously^6^. Primers were designed using the NCBI primer-blast tool combining Primer3 and BLAST to find amplicon specific primers^56^. Sequences of each primer pair are listed in Supplementary Table 3. Threshold cycle (CT) values for all selected genes were normalized to the CT value of three bluegreen aphid reference genes, an elongation factor 1 gene, an actin gene and the ribosomal L27 gene, whose expression remained constant in aphids among various treatments. Two technical repeats and three biological repeats (with one repeat consisting of five cages per treatment) were used for data analyses. The significance in difference between ratios was analyzed using analysis of variance (ANOVA) and Bonferroni post-hoc multiple comparison testing to determine which treatments are significantly different at a 1% significance level (*P* < 0.01) using the GraphPad Prism Statistical Software program.

### Ethical approval

This article does not contain any studies with human participants performed by any of the authors.

## Supplementary information

Supplementary Data File.
