# Supplementary material for: An RNAi supplemented diet as a reverse genetics tool to control bluegreen aphid, a major pest of legumes
Source: Sci Rep. 2020 Jan 31;10:1604. doi: 10.1038/s41598-020-58442-4 (PMC6994723; doi:10.1038/s41598-020-58442-4)
Supplement: Supplementary file 1 — Supplementary Data File. [file 41598_2020_58442_MOESM1_ESM.pdf]

An RNAi supplemented diet as a reverse genetics tool to control bluegreen aphid, a major pest of legumes.

Silke Jacques[1,2], Jenny Reidy-Crofts[1], Jana Sperschneider[3], Lars G. Kamphuis[1,2], Ling-Ling Gao[1], Owain R. Edwards[4], Karam B. Singh[1,2*]

[1] Centre for Environment and Life Sciences, CSIRO Agriculture and Food, Floreat, WA 6014, Australia

[2] Curtin University, Centre for Crop and Disease Management, Bentley, WA 6102, Australia

[3] Biological Data Science Institute, The Australian National University, Canberra, ACT 2600, Australia

[4] Centre for Environment and Life Sciences, CSIRO Land and Water, Floreat, WA 6014, Australia

[*] Author for correspondence: karam.singh@csiro.au; phone: +61 8 9333 6320; fax: +61 8 9387 8991

Supplementary Data File

Supplementary Figure

Supplementary Figure 1

```
>AkC002
AAAACACTTGTGCAATACCGTTTCACAAAGTATAAAACCTAGTAATTTTTTATCAAAAGT
TTGTTCAAACAAATATCTCGTCGTGTATCCAGTGCGATAGCGATTATTTACAACATGGGA
AGTTACAAATTATACGTAGCCGTCATGGCAATAGCCATAGCTGTAGTACAGGAAGCTAGT
TGCGGTGATTGGTCTGACACTGAACAGTACGATGAGCAGGAAGAAGCCTTTGTCGAATTA
CCGTCAGTGGAGCACCGTCAGTGCGATGAATACAAATCGAAGATCTGGGACAAAGCATTT
AGCAACCAGGCGGCTATGCAACTGATGGAACTAATGTTTATTACAGGTAAGGAATTAGGC
TCCAACGAAGTGTGCTCGGACACGACGCGGGCCATTTTTAACTTCGTCGATGTGATGGCC
ACCAACCAGAACGCCCATTACTCGCTGGGTATGATGAACAAGATGCTGTCGTTCATCGTG
AGGGAGGTGGACACGACGTCCGACAAATTCAAAGAGACGAAGGCGGTTGTCGAACGCATC
GCGAAAAATCCAGAGATCCGAGACTATATCAAGCACACGACCGCCAGGGCCGTCGACTTG
CTCAAGGAGCCCGTTATTAGAGGCCGACTGTTCAAAGTGATGAAAGCTTTCGAGGGTCTG
ATAAAACCATCTGAAAACGAGGAATTGGTCAAGCAGAGGATTAAGAGGCTAACCAATGCT
CCCGCCAAGATGGCTATGGGAGCCATGAATAAGGTTGGAAGTTTCTTACGACGTTTTTAA
TAAGCGCGTCCATACAGACTAGTGATATACTTATAGATAAATATAAAGTGCAGATTTTTT
AAAAAAAATTTGAAATCTCACTCGCCTACCGCTTCGATAGTCGTCTTCAGTTTTTTGTAC
ATTGGGTACAATTACACATTAGGTATATTTGATTCACCACTCATTACGTTGTCCTTATAA
ATACTATCGTTATTGGTATTATTATTATTATTATTATTA

>Akhbd
ATGTTTTTGGAAACGAACACCAGCCAATTATTAGCCATTCACCTTACGACCTAGCACGTC
CACTAGAACACCATCACCATCATCACCTCAGCCATCACGCTGCGTCCAATCATCTCCAGT
CGAGCACCGATCCCTTGCAAGAAGATTCAGAAGGTCGGAGTACAGCTAGCTCGCCGACGT
CCAGTTACCGCGACCTATCCGGGTGCGAAGACTTTGATCTGAAAATCCCGAAACCGTCTG
GTCACGGGTCTGGCACTGGTGCGAATAACAACAATAACCACAATAACAACAACAACAAAG
AACAAAAACCGAAGAAGCACAAGTGCAAACACTGTGGGCTGGAGTGCACGGAAAAGGTGC
AGTACTGGAAGCACATTCGCACTCACATCAAACCTGAACAGTTGCTTGAGTGTCCAAACT
GTGAGTTCGCCACCGACCTGAAACACCACTACGAATACCACCTGCTGAACCACACGGGCG
CCAAACCGTTCACGTGCCCGGACTGCGACTACAAGTGCGTGAGCAAGTCGATGCTCCAGT
CGCACCTCAAGTCGCATTCGAACGTGTTCCAGTTCCAGTGTTACGACTGTGGTTACGCAT
CTAAGTACATGCACAGCCTCAAGCAGCACCTGAAGAAGCGCGACCACCGGCCGGCCACGC
CGCTCAACCCAGACGGCACGCCGAACCCGGACATCGTCATCGACGTGGTAGGCAACCGAC
GCGGGCCGCGGCAGAACAAGAAACAAAACCGGCACAACCCGTATCAGCAACAGCACCAGC
AGCAACAGCTCCAGCAGTCTATGACGGTGTGTTCGTCGCAGGACGACGGCGGCAGCAGTA
GCGGCGGCCACCCATCCCCGTACTCGATGCAACAGCTGTTGCAGATGCCCGCTTCCGGAT
GCGCCCAGGTCACGTATCCGACTTCGGCCGAGTCGTTCATGTATTCGATGATCACCAAAC
GCTCCATAGAGATGGTTGACTACCAGGCCGCCGAATACATGGCCATCAAGAATAACAACA
ACAACAATAATAACAACTCCTCGTCGGTCGACGATAAGATGGAAGTGCAACAGCACCACC
AACAACCACTACAACACCACCACAACAATCATCACCATAAAACGCGGCCATCAGTACCAT
TCAGATGACGTGCTGAAAGTGGACTTGGCTGGGCGTAGCGGTAGTGGCGGTGAATTGACG
CCCGAACCACAGGTACCTGTGTTGGCGGTCGCCGATCCTTCGCCTCCGACCGTCGTTTTG
GCCATCGAGACGGGACCGTTGAACTTAAGCAGGGACTCGGTGGCTCCTCGCGCCGCGGGC
AGCAGCCGGCGCAAGGGGATCGCGTGCAAACTCGAGCGACCGGCGACCGAATCGCAGCCC
AAGTCGGTGCCGGTGCCGGTGGTGGTTGTTCCGGTGTCGCCTACGGTACCGATGGATTGC
AGTAGCGAGTCAAGAGGAATGGTGGAGGAGACTTTGATTCCGGTAAAGGAAGAATATCAA
CCGTACCACCACTACCATCATCATCAGCAGCAGCAGCAGCAGCAGCAGACGTCATCGTTG
TCTTCGACGCCTGAAAAAGAAGACAAGGAAGACGAGACGCACGTGTGTCACCACTGCGAC
ATAATATTCAAGGAGAACATTATGTACTCGATGCACATGGGTTTCCACAGCTTCAGGGAC
CCGTTCGCGTGCAACTTGTGCGGCGAGATAACGGCAGATAAGTTTTCGTTTTTCGCTCAC
ATAGCGCGCGTACCGCACAGTTAGGAGGTGCACCTACGAAAGATACGAAATAATAATAGA
GTACAAATTTTAAAACAATTAACCCTTTA
```

Supplementary Figure 1: bluegreen aphid sequences of the C002 and Aphbd homologous genes

The pea aphid salivary gland and hunchback gene sequences were blasted against the genome sequence of bluegreen aphid available in-house. The obtained sequences of the *AkC002* and *Akhbd* are shown and the primers targeting conserved regions are highlighted in yellow.

Supplementary Figure 2

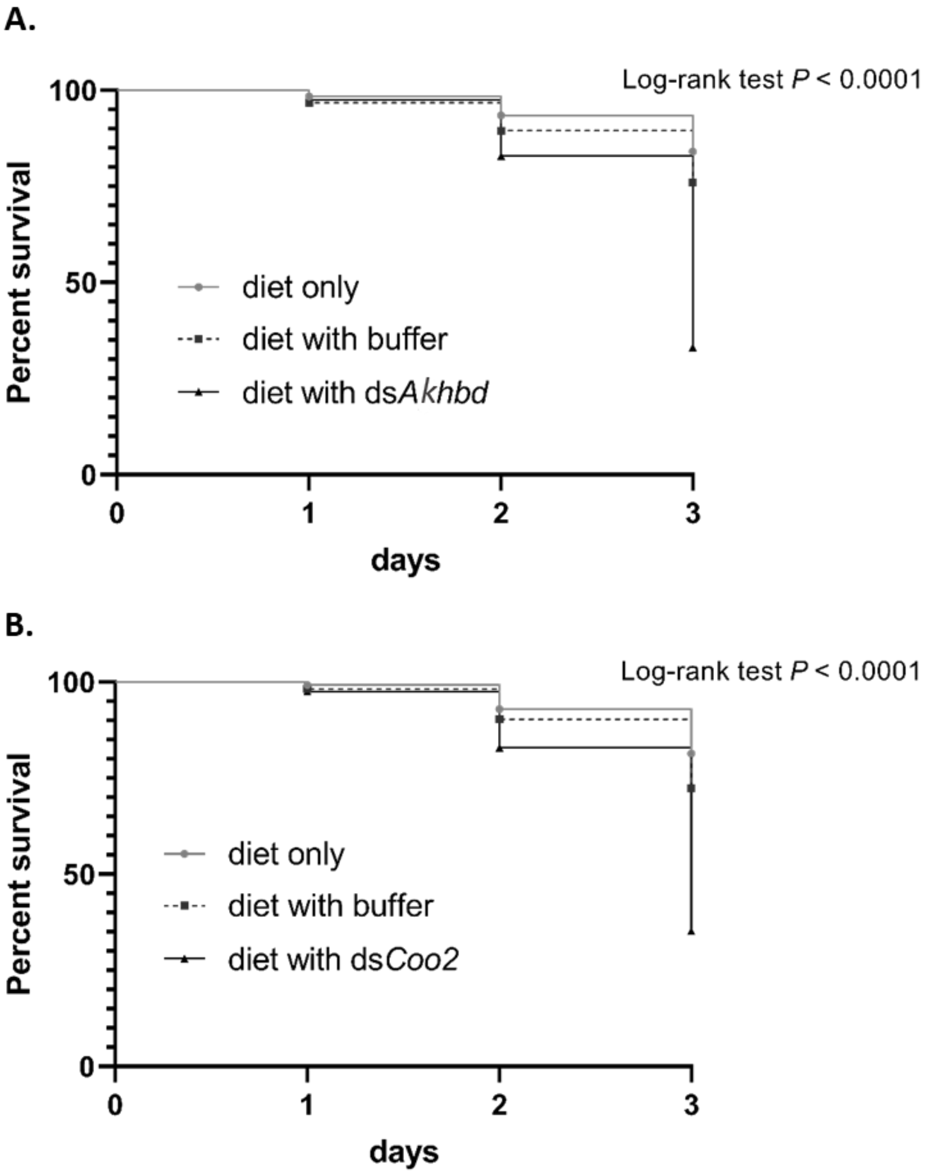

Supplementary Figure 2: Kaplan-Meier survival curves show highly significant differences between diet treatments.

Bluegreen aphid survival curves are displayed as percent survivors over three days whilst feeding on diet only (circle, light grey line), diet mixed with elution buffer (square, dotted line), or diet supplemented with dsRNA (triangle, black line) either targeting *Akhbd* (A) or *C002* (B). The Kaplan-Meier log ranking omnibus test shows a significant difference in survival curves due to diet treatment ($P < 0.0001$).

Supplementary Tables

Supplementary Table 1

Nr of aphids per cage: 5

Timeframe: 48 hrs

DADD + MITTLER - 15% sucrose					
cage	*Nr of survivors*	*total weight - on*	*mean weight -on*	*total weight - off*	*mean weight - off survivors*
1	0	0.27	0.054		
2	1	0.23	0.046	0.064	0.064
3	5	0.24	0.048	0.4	0.08
4	4	0.204	0.041	0.349	0.087
5	2	0.193	0.039	0.127	0.064
Mean	**2.4**	0.227	0.046	0.235	0.074
SE	0.69	0.01	0.002	0.055	0.004

DADD + MITTLER - 17.5% sucrose					
cage	*Nr of survivors*	*total weight - on*	*mean weight -on*	*total weight - off*	*mean weight - off survivors*
1	5	0.282	0.056	0.561	0.112
2	3	0.263	0.053	0.239	0.079
3	4	0.272	0.054	0.358	0.089
4	3	0.242	0.048	0.173	0.058
5	5	0.234	0.047	0.443	0.089
Mean	**4**	0.259	0.052	0.355	0.085
SE	0.45	0.009	0.002	0.069	0.009

Supplementary Table 1: Bluegreen aphid survival rate on diet with increased sucrose levels

Bluegreen aphid survivor rate on a synthetic diet formulated for green peach aphid [10] was less than 50 % after 48 hrs. By increasing the sucrose concentration to 17.5 %, the bluegreen aphid survivors increased to 80 % after two days.

Supplementary Table 2

Amino Acids	Amount (mg)	Vitamins	Amount (mg)
Alanine	100	Ascorbic acid	100
Arginine	270	Biotin	0.1
Asparagine	550	Pantothenic acid	5
Aspartic acid	140	Choline chloride salt	50
Cysteine HCl	40	Folic acid	0.5
Glutamic acid	140	Myo-inositol	50
Glutamine	150	Nicotinic acid	10
Glycine	80	Pyridoxine	2.5
Histidine	80	Riboflavin	0.5
Isoleucine	80	Thiamine HCl	2.5
Leucine	80	**Other**	
Lysine HCl	120	Potassium dihydrogen orthophosphate	500
Methionine	40	Magnesium chloride hexahydrate	200
Phenylalanine	40	Cupric-Sodium EDTA salt	0.4
Proline	80	Ferric-Sodium EDTA salt	1.5
Serine	80	**Manganese chloride tetrahydrate** *	0.4
Threonine	140	Zinc Chloride	0.8
Tryptophan	80	**Cholesterol** *	2.5
Tyrosine	40		**Amount (g)**
Valine	80	**Sucrose** *	17.5
			**Amount (mL)**
		Sterile Water	100

Supplementary Table 2: bluegreen aphid artificial diet composition

The bluegreen aphid artificial diet was based on the previously described diet for *Myzus persicae* [10]. Supplementary Table 2 summarizes the compounds and their respective amounts to make 100 mL of artificial diet. Changes to the original Dadd & Mittler diet are marked by an asterisk and highlighted in red.

Supplementary Table 3

Gene	Forward primer	Reverse primer
Akhbd	5'-AAGCACATTCGCACTCACA-3`	5'-GTTCAGCAGGTGGTATTCGT-3`
C002	5'-AGGAAGAAGCGTCTGTCGAA-3'	5'-AGTAATGGGCGTTCTGGTTG-3`
EF1 (Elongation Factor 1)	5'-CTGTGCTTATTGTCGCTGCT-3'	5'-TCGCTGTATGGTGGTTCAGT-3`
ACTIN	5'-CAATGGGACAGATTAGGTAG-3'	5'-AGCATCCGACAAAGTAGC-3`
L27	5'-CCGAAAAGCTGTCATAATGAAGAC-3'	5'-GGTGAAACCTTGTCTACTGTTACATCTTG-3`

Supplementary Table 3: Primer pairs used for qRT-PCR amplification of target genes (Akhbd and C002) and housekeeping genes (EF1, ACTIN and L27).
